# Neurobiological Mechanisms of Transcranial Direct Current Stimulation for Psychiatric Disorders; Neurophysiological, Chemical, and Anatomical Considerations

**DOI:** 10.3389/fnhum.2021.631838

**Published:** 2021-02-04

**Authors:** Yuji Yamada, Tomiki Sumiyoshi

**Affiliations:** ^1^Department of Psychiatry, National Center Hospital, National Center of Neurology and Psychiatry, Tokyo, Japan; ^2^Department of Preventive Intervention for Psychiatric Disorders, National Institute of Mental Health, National Center of Neurology and Psychiatry, Tokyo, Japan

**Keywords:** transcranial direct current stimulation, non-invasive brain stimulation, neurotransmitter, LTP, neuromodulation, neural network

## Abstract

**Backgrounds:** Transcranial direct current stimulation (tDCS) is a non-invasive brain stimulation technique for the treatment of several psychiatric disorders, e.g., mood disorders and schizophrenia. Therapeutic effects of tDCS are suggested to be produced by bi-directional changes in cortical activities, i.e., increased/decreased cortical excitability via anodal/cathodal stimulation. Although tDCS provides a promising approach for the treatment of psychiatric disorders, its neurobiological mechanisms remain to be explored.

**Objectives:** To review recent findings from neurophysiological, chemical, and brain-network studies, and consider how tDCS ameliorates psychiatric conditions.

**Findings:** Enhancement of excitatory synaptic transmissions through anodal tDCS stimulation is likely to facilitate glutamate transmission and suppress gamma-aminobutyric acid transmission in the cortex. On the other hand, it positively or negatively modulates the activities of dopamine, serotonin, and acetylcholine transmissions in the central nervous system. These neural events by tDCS may change the balance between excitatory and inhibitory inputs. Specifically, multi-session tDCS is thought to promote/regulate information processing efficiency in the cerebral cortical circuit, which induces long-term potentiation (LTP) by synthesizing various proteins.

**Conclusions:** This review will help understand putative mechanisms underlying the clinical benefits of tDCS from the perspective of neurotransmitters, network dynamics, intracellular events, and related modalities of the brain function.

## Introduction

**Figure 1:**
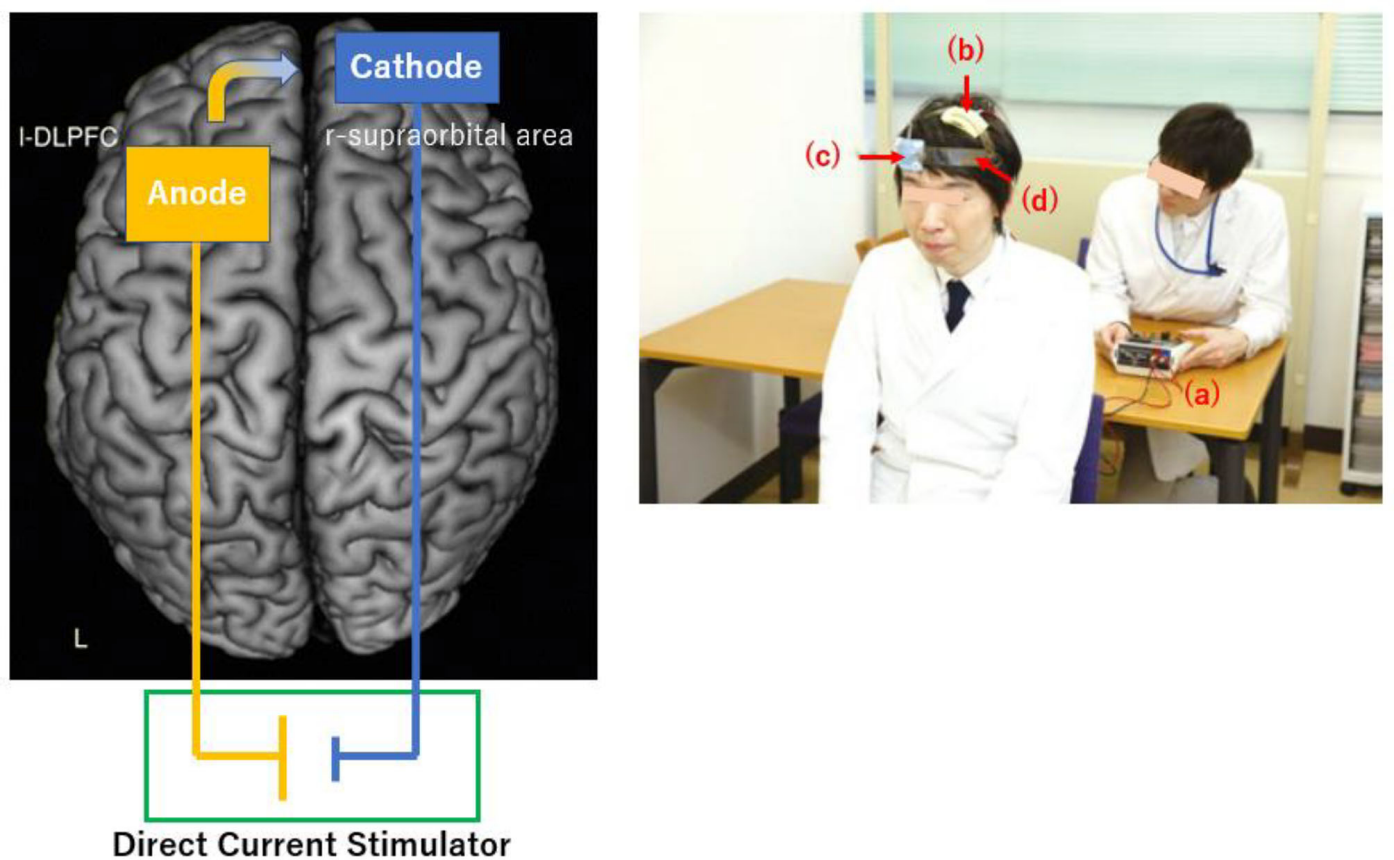
Schematic diagram **(Left)** and experimental setup **(Right)** for tDCS. **(Left)** The anode and cathode electrodes are positioned over the left dorsolateral prefrontal cortex and over right supraorbital region, respectively. The direction of current flow is from the anode to cathode. **(Right)** An administrator controls the stimulator (a). Anodal (b) and cathodal (c) electrodes of 35-cm^2^ in size are placed on F3 and right supraorbital region, respectively. A head strap (d) is used as needed to increase reproducibility.

Transcranial direct current stimulation (tDCS) is a non-invasive method that modulates neural activities in the brain by delivering low-amplitude (usually no more than 2 mA) over a short period (no more than 30 min) between electrodes (anode and cathode). At least, one of the electrodes is placed on the scalp, through which electronic currents penetrate the skull to enter the brain and facilitate or inhibit spontaneous neural activities in the vicinity of electrodes (Yokoi et al., 2018; Figure 1).

Effectiveness of tDCS in the treatment of major depressive disorder (MDD) has been reported (Yokoi et al., 2018). Thus, a meta-analysis has shown a moderate effect of tDCS on depressive symptoms in patients with acute depression (Hedges'g = 0.37) (Shiozawa et al., 2014). Also, there has been a series of reports showing the ability of tDCS to ameliorate positive/negative symptoms of schizophrenia (Kim et al., 2019). For example, hallucinations (positive symptoms) (Hedges'g = 0.86) and negative symptoms (0.41) have been found to be improved by multi-session tDCS on the frontal or frontotemporal lobe (see montages in Table 1) twice daily for 5 days (Kim et al., 2019). Moreover, meta-analysis for cognitive function in patients with schizophrenia indicates the ability of multi-session tDCS on the prefrontal cortex (see montage in Table 1) to improve working memory (Hedges'g = 0.49), an important cognitive domain (Narita et al., 2020; Table 1).

**Table 1:** Meta-analyses of the effects of tDCS.

**Study**	**Target disease**	**No. of RCT (*n*)**	**Montage (anode/cathode)**	**Intensity (mA)**	**Duration (min)**	**No. of sessions**	**Outcomes**	**Effect size (Hedges'g)**
Shiozawa et al. (2014)	MDD	7 RCTs (259)	F3/RSO, F3/F4, F3/F8	1–2	20–30	5–15	HAMD MADRS	0.37
Kim et al. (2019)	Schizophrenia (positive symptoms)	5 RCTs (186)	Between F3 and FP1/Between T3 and P3	2	20	10–15	AHRS PANSS PSYRATS	0.86
Kim et al. (2019)	Schizophrenia (negative symptoms)	7 RCTs (257)	Between F3 and FP1/Between T3 and P3, F3/F4	2	20–30	10–15	PANSS SANS	0.41
Narita et al. (2020)	Schizophrenia (cognitive functions)	9 RCTs (270)	Between F3 and FP1/Between T3 and P3, F3/F4, F3/FP2	1–2	20–30	2–40	Digit span MCCB, SOPT N-back task 2-back task	0.49 (working memory)

*MDD, major depressive disorder; RCT, randomized controlled trial; HAMD, Hamilton Rating Scale for Depression; MADRS, Montgomery-Åsberg Depression Rating Scale; AHRS, Auditory Hallucination Rating Scale; PANSS, Positive and Negative Syndrome Scale; PSYRATS, Psychotic Symptom Rating Scales; SANS, Scale for the Assessment of Negative Symptoms; MCCB, MATRICS Consensus Cognitive Battery; SOPT, Self-Ordered Pointing Task*.

In spite of accumulated evidence for the efficacy of tDCS in treating psychiatric disorders, particularly schizophrenia and mood disorders, its mechanism of action has not been fully elucidated (Stagg and Nitsche, 2011). Therefore, the current review aimed to provide an overview of the actions of tDCS, especially anodal stimulation, on neurotransmission and neural networks in the brain, to help understand the mechanisms underlying its therapeutic effects.

The effect of tDCS on psychiatric symptoms has been mainly reported in studies using anodal stimulation over the frontal cortex. On the other hand, where the cathodal electrode is placed has not been uniform, indicating anodal stimulation has attracted interests to consider the mechanism of tDCS (Fregni et al., 2020). As the clinical benefits of tDCS have been found when multi-sessions are applied (Shiozawa et al., 2014; Kim et al., 2019; Narita et al., 2020), emphasis was placed on long-term changes of neural events produced by tDCS.

## Neurophysiological Understanding of tDCS

**Figure 2:**
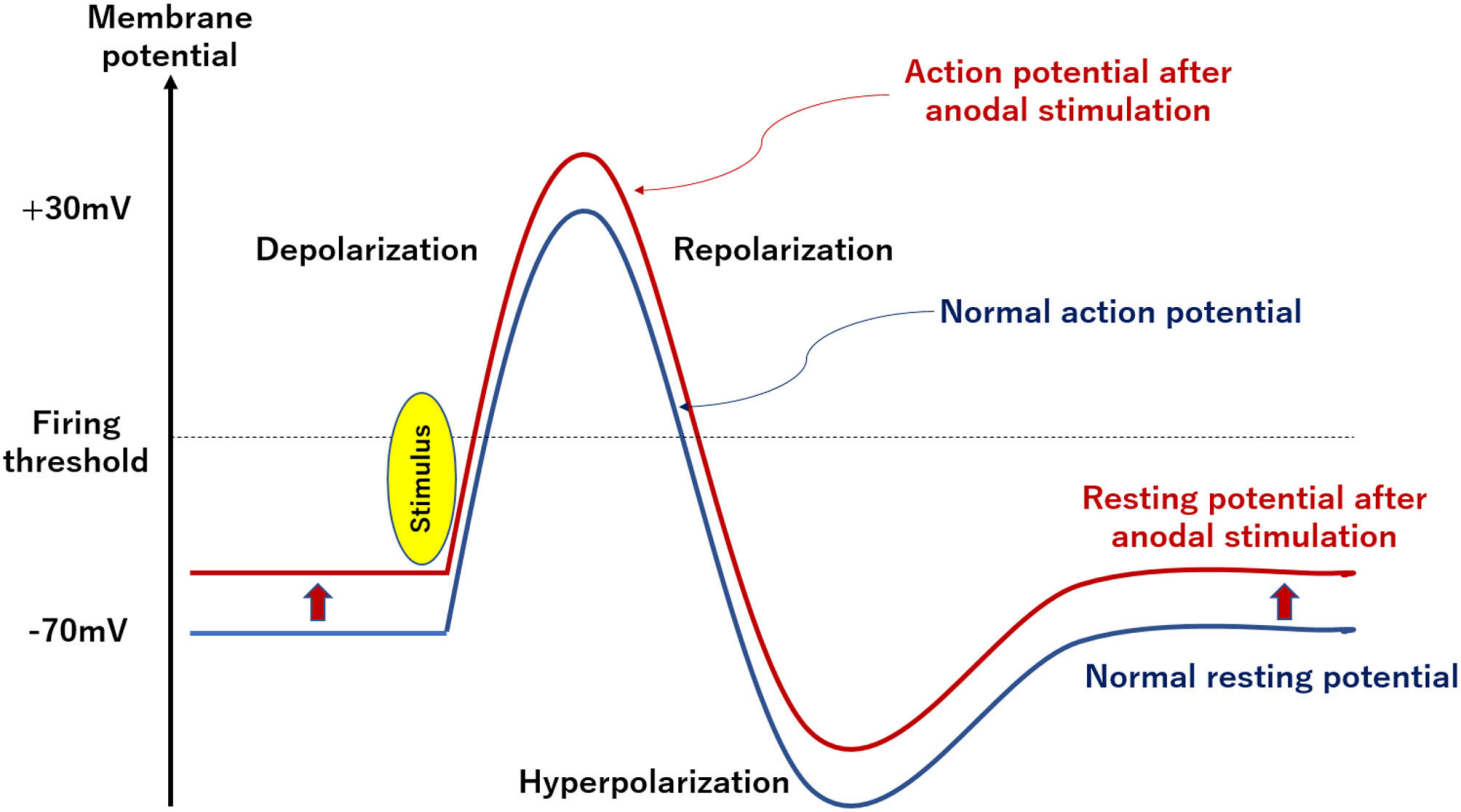
Schematic diagram of changes in neuron potential by tDCS. Anodal stimulation with tDCS (1–2 mA) is not strong enough to depolarize the membrane potential of neurons to the firing threshold. Conversely, cathodal stimulation is thought to deepen the resting membrane potential and make it difficult for neurons to depolarize.

Anodal stimulation with tDCS (1–2 mA) by itself is not strong enough to depolarize the membrane potential of neurons to the firing threshold, and only increases the rate of spontaneous combustion and their excitability (Nitsche and Paulus, 2000; Philip et al., 2017; Figure 2). Conversely, cathodal stimulation is thought to deepen the resting membrane potential, making it difficult for neurons to depolarize, which reduces spontaneous combustion rates and excitability of neurons (Nitsche and Paulus, 2000; Philip et al., 2017; Figure 2). Importantly, these effects of tDCS depend on the intensity and duration of stimulation (Nitsche and Paulus, 2000), and radial electric field (Seo and Jun, 2019).

Electrophysiological understanding of tDCS may be facilitated by the stimulation-dependent model (Fertonani et al., 2011). In this model, anodal stimulation is considered to promote depolarization of neurons, and cathodal one causes hyperpolarization to suppress it. Moreover, electrical stimulation affects multiple neurons and increases their membrane potentials to induce depolarization. These events in the vicinity of neural membranes has been proposed to explain the ability of tDCS to improve brain functions (Silvanto et al., 2008).

Increased excitability of local neurons by anodal stimulation is thought to increase blood flow around the stimulation site, and induce subsequent metabolic changes. Specifically, blood-flow changes through tDCS on the prefrontal cortex have been measured by functional near-infrared spectroscopy (fNIRS) (Merzagora et al., 2010). In this study, the increase of oxygenated hemoglobin concentrations under the anodal electrode was significantly larger than those for the cathode. This is thought to reflect the ability of anodal stimulation to induce metabolic changes among neurons (Merzagora et al., 2010).

## Biochemical Effects of tDCS

Changes in neurotransmissions by anodal stimulation have been reported in relation to metabolic changes in the brain. Here, we review the accumulated evidence for the effect of stimulation of the motor cortex in patients with chronic pain and those receiving post-stroke motor rehabilitation (Medeiros et al., 2012; Tables 2, 3). For example, the effect of anodal stimulation is suppressed by carbamazepine (sodium channel inhibitor) (Liebetanz et al., 2002), indicating that inhibition of intracellular influx of extracellular sodium ion suppresses anode-induced depolarization of neurons, and subsequent excitements.

**Table 2:** Effects of concomitant medication on anodal tDCS on the motor cortex of healthy subjects (adapted from Medeiros et al., 2012).

**Study**	***N***	**Stimulation intensity (mA)**	**Stimulation duration (min)**	**Stimulation site**	**Pharmacological intervention**	**Effects**
Liebetanz et al. (2002)	11	1	5	Left M1	Carbamazepine (CBZ) Dextromethorphan (DMO)	Both drugs suppressed the effect of tDCS.
Nitsche et al. (2003)	11–14	1	11~13	Left M1	CBZ, DMO Flunarizine (FLU)	All suppressed the effect of tDCS.
Nitsche et al. (2004a)	6–12	1	11	Left M1	Lorazepam (LOR)	Delayed the effect of tDCS.
Nitsche et al. (2004b)	12	1	13	Left M1	d-cycloserine (d-CYC)	Prolonged the effect of tDCS.
Nitsche et al. (2004c)	5–12	1	13	–	Amfetaminil (AMP) Propranolol (PRO)	AMP enhanced and prolonged the effect of tDCS while PRO shortened it.
Nitsche et al. (2006)	4–12	1	13	Left M1	Sulpiride	Suppressed and delayed the effect of tDCS.
Kuo et al. (2007)	10–12	1	13	Left M1	Rivastigmine (RIVA)	Suppressed the effect of tDCS.
Kuo et al. (2008)	7–11	1	13	Left M1	Levodopa (L-dopa)	Suppressed the non-specific effects of tDCS while enhanced local effects on synapses of specific neurons.
Rango et al. (2008)	10	1.5	15	Right M1	None	Increased the myo-inositol content under the anode electrode.
Nitsche et al. (2009)	12	1	13	–	Citalopram (CIT)	Enhanced and prolonged the effect of tDCS.
Stagg et al. (2009)	7–11	1	10	–	None	Locally decreased GABA in the cortex. Glutamic acid decreased in correlation with the decrease in GABA due to cathodal stimulation.
Monte-Silva et al. (2010)	12	1	13	Left M1	L-dopa	Suppressed the effect of tDCS.
Stagg et al. (2011)	12	1	10	Left M1	None	Decreased GABA. Positive correlation was found between motor learning and changes in the fMRI signal on the left M1.
Thirugnanasambandam et al. (2011)	48	1	13	Left M1	Nicotine	Suppressed the effect of tDCS.
Chaieb et al. (2012)	8	1	5	Left M1	d-CYC	Suppressed the effect of tDCS.

*tDCS, transcranial direct current stimulation; M1, primary motor cortex*.

**Table 3:** Pharmacological actions.

**Drug**	**Pharmacological action**
Carbamazepine (CBZ)	Sodium channel inhibitor
Dextromethorphan (DMO)	NMDA receptor inhibitor
Flunarizine (FLU)	Calcium channel inhibitor
Lorazepam (LOR)	GABA receptor agonist
d-cycloserine (d -CYC)	NMDA receptor partial agonist
Amfetaminil (AMP)	Adrenergic receptor agonist
Propranolol (PRO)	Adrenergic receptor inhibitor
Sulpiride (SUL)	Dopamine receptor inhibitor
Pergolide (PGL)	Dopamine receptor agonist
Rivastigmine (RIVA)	Cholinesterase inhibitor
Levodopa (L-dopa)	Dopamine precursor
Citalopram (CIT)	Serotonin reuptake inhibitor
Nicotine	Nicotinic acetylcholine receptor agonist

Glutamate receptor subtypes governing excitatory synaptic transmissions include AMPA (α-amino-3-hydroxy-5-methyl-4-isoxazolepropionic acid) and NMDA (N-methyl-D-aspartate) receptors, both of which are coupled with ion-channels. The AMPA receptor is involved in the intracellular influx of sodium ion during neuronal depolarization, causes transient action potentials, and accounts for most of the excitatory synaptic transmissions. On the other hand, the NMDA receptor is involved in the intracellular influx of calcium ion during depolarization, produces prolonged action potentials, and mediates neural circuits governing memory and learning. Therefore, actions on NMDA receptors, inducing plasticity of neurons, play a dominant role in improving symptoms of psychiatric disorders. Accordingly, dextromethorphan (NMDA receptor inhibitor) suppresses the effect of anodal stimulation (Liebetanz et al., 2002; Nitsche et al., 2003, 2004a), while d-cyclo-serine (partial NMDA receptor agonist) prolongs it (Nitsche et al., 2004b). This is in line with the observations that NMDA receptor agonists enhance excitatory synaptic transmissions, while NMDA receptor inhibitors suppress it (Liebetanz et al., 2002; Nitsche et al., 2003, 2004a,b). Also, GABA (gamma-aminobutyric acid: γ-aminobutyric acid), a neurotransmitter that inhibits synaptic transmissions, may play a role. Thus, lorazepam, a GABA receptor agonist, delays the effect of anodal stimulation (Nitsche et al., 2004c). On the other hand, the anodal stimulus itself causes a local decrease in GABA concentrations in the cortex (Stagg et al., 2009, 2011).

Monoamine neurotransmitters, such as dopamine, serotonin, and acetylcholine have been reported to mediate the effect of tDCS (Nitsche et al., 2006, 2009; Kuo et al., 2007, 2008; Monte-Silva et al., 2010; Thirugnanasambandam et al., 2011). For example, sulpiride, a dopamine receptor blocker (Nitsche et al., 2006), suppresses the effects of anodal stimuli, while levodopa, a dopamine precursor (Kuo et al., 2008; Monte-Silva et al., 2010), locally enhances excitement of certain synaptic transmissions (Kuo et al., 2008). These findings suggest that the action of tDCS may include regulation of dopamine transmissions. Also, citalopram, a serotonin reuptake inhibitor, enhances anodal stimulation (Nitsche et al., 2009). Regarding acetylcholine transmissions, rivastigmine, a cholinesterase inhibitor, suppresses the effect of tDCS (Kuo et al., 2007). In sum, the direction of influence on actions of anodal stimulation varies depending on monoamine neurotransmitters.

The above considerations overall lead to the concept that anodal stimulation enhances excitatory synaptic transmissions by stimulating glutamate transmissions and suppressing GABA transmissions in the cortex. On the other hand, it modulates the dopamine system, enhances and suppresses serotonin and acetylcholine transmissions, respectively. These effects of tDCS on monoamine transmissions are considered to be associated with change of the balance between excitatory and inhibitory inputs in the brain (Okun and Lampl, 2008).

## Neuroanatomical Understanding of the Effect of tDCS

Impaired functional connectivity between brain regions has been reported in patients with psychiatric disorders, such as schizophrenia and bipolar disorder (Yamada et al., 2020). In schizophrenia patients, a study using resting functional magnetic resonance imaging (fMRI) found a separation between the medial prefrontal cortex and the dorsolateral prefrontal cortex (Chai et al., 2011). Another study found changes of dynamic functional connectivity mainly in the thalamus and cerebellum, as well as frontal, temporal, occipital, fusiform, post-central, cuneus, supramarginal, and calcarine cortices in patients with schizophrenia or bipolar disorder. Specifically, functional connectivities involving the post-central, frontal, and cerebellar cortices are weakened across schizophrenia and bipolar disorder, while those involving the insular, temporal, frontal, fusiform, lingual, occipital, supramarginal cortices, as well as thalamus and cerebellum, are strengthened (Du et al., 2017). Kunze et al. systematically applied tDCS to a large-scale network model consisting of 74 brain regions to investigate the functional connectivity of dynamic states. They found alterations of the competitive interrelationship of functional networks by tDCS (Kunze et al., 2016).

Based on these findings, the mechanism of action of tDCS on neural circuits are summarized in Table 4. Anodal tDCS may enhance excitatory synaptic transmissions by changing the balance between glutamate and GABA activities (Clark et al., 2011; Stagg et al., 2014; Bachtiar et al., 2015; Hunter et al., 2015), leading to modification of functional connectivity between brain regions, including the stimulation site (Polanía et al., 2011; Stagg et al., 2014; Bachtiar et al., 2015; Hunter et al., 2015). Furthermore, the effects of tDCS may be extended in the brain, through an increased/decreased release of monoamine transmitters, such as dopamine, on neural circuits that do not necessarily involve the anodal stimulation site (Polanía et al., 2011; Hunter et al., 2015; Fonteneau et al., 2018). These neural events are thought to improve psychiatric symptoms and cognitive function (Fukai et al., 2019). In summary, anodal stimulation is likely to modify activity levels of both specific brain regions and multiple network systems (Luft et al., 2014).

**Table 4:** Changes in the brain networks by anodal tDCS.

**Study**	**Subject (*n*)**	**Stimulation intensity (mA)**	**Stimulation duration (min)**	**Stimulation site**	**Pharmacological intervention**	**Results**
Clark et al. (2011)	Healthy subjects (7)	2	30	Right parietal lobe	None	Increased glutamic acid concentrations under anode electrodes.
Polanía et al. (2011)	Healthy subjects (13)	1	10	Left primary motor cortex	None	Reduced direct functional connectivity to gray matter away from the left somatomotor cortex (SM1). Enhanced functional connectivity between the premotor area and the parietal lobe via the left SM1. Enhanced functional connectivity between the left posterior cingulate cortex and the right DLPFC.
Stagg et al. (2014)	Healthy subjects (10)	1	10	Left primary motor cortex	None	Negative correlation between GABA concentrations and functional connectivity of the resting motor network. Decreased GABA concentrations. Enhanced the functional connectivity of the resting motor network.
Hunter et al. (2015)	Healthy subjects (9)	2	30	Right parietal lobe	None	Increased glutamic acid concentrations under anode electrodes. Enhanced the functional connectivity of the superior parietal-inferior parietal-left frontal parietal-cerebellum. Suppressed the functional connectivity of the anterior cingulate-basal ganglia.
Bachtiar et al. (2015)	Healthy subjects (12)	1	20	Left primary motor cortex	None	Decreased GABA concentrations. Enhanced the functional connectivity of the resting motor network. Decreased GABA concentrations and enhanced functional connectivity of motor networks by different mechanisms.
Fonteneau et al. (2018)	Healthy subjects (32)	2	20	Both dorsolateral prefrontal cortex (DLPFC)	None	Increased dopamine release in the striatum.
Wörsching et al. (2018)	Healthy subjects (28)	2	20	Right DLPFC	None	Decreased resting-state fMRI connectivity in a medial part of the left prefrontal cortex. Decreased regional brain activity during a delayed-response working-memory (DWM) retrieval. Faster responses to the DWM task.
Fukai et al. (2019)	Healthy subjects (20)	2	26	Left DLPFC	None	Increased dopamine release in the right ventral striatum.
Bulubas et al. (2019)	Patients with major depression (52)	2	30 (22 sessions)	Left DLPFC	Escitalopram 20 mg/day	Association between larger gray matter volumes and depression improvement assessed over a treatment period of 10 weeks.

## Mechanism of Action of tDCS via Long-Term Potentiation and Glial Cells

**Figure 3:**
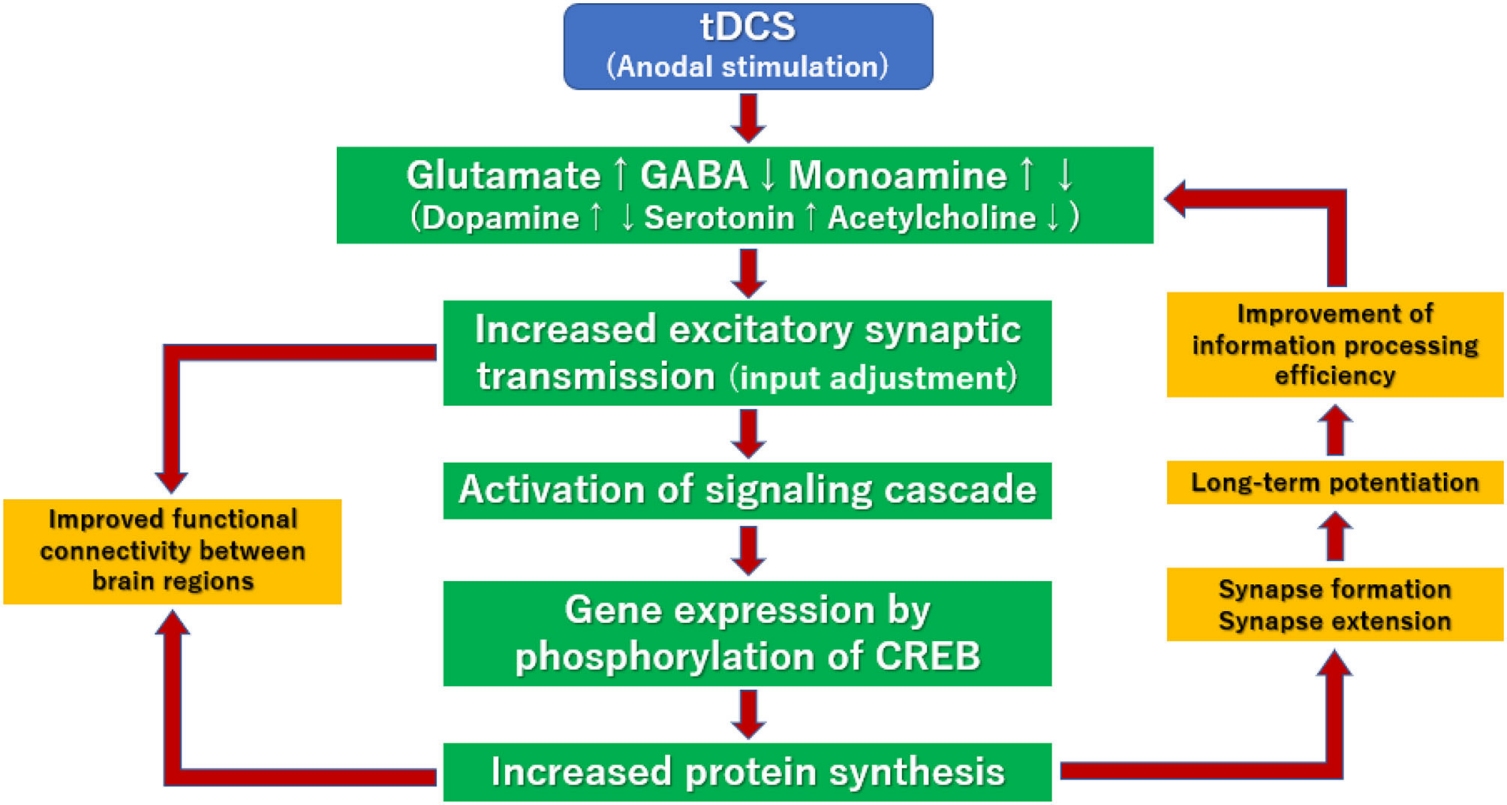
Putative mechanisms for the enhancement of long-term potentiation by tDCS. Various neurotransmitters activate/inhibit transduction cascades bound to G-proteins or ion-channels, leading to phosphorylation of cAMP-responsive element binding protein (CREB) and activation of genes in the nucleus of neurons. These signal transduction cascades enhance the synthesis of various proteins, such as neurotransmitter synthases, receptors, ion channels, and intracellular signal proteins. Facilitative actions of these proteins that regulate efficiency of neurotransmissions in the cerebral cortex circuit may explain the ability of tDCS to induce LTP.

Long-term potentiation (LTP), continuous enhancement of signal transduction between neurons, is thought to mediate the effect of tDCS (Figure 3). First, action potentials in presynaptic neurons are converted into chemical signals at the presynaptic membrane. Subsequently, neurotransmitters (glutamate, GABA, dopamine, serotonin, acetylcholine, etc.) are released into the synaptic gap. The process by which this neurotransmitter is transmitted to post-synaptic neurons is called the signal transduction cascade. In this cascade, various neurotransmitters activate/inhibit transduction cascades bound to G-proteins or ion-channels, leading to phosphorylation of cAMP-responsive element binding protein (CREB) and activation of genes in the nucleus of neurons. Also, the neurotrophic factor-bound transduction cascade may play a role by activating various kinase enzymes (Stephen, 2013). These signal transduction cascades enhance the synthesis of various proteins, such as neurotransmitter synthases, receptors, ion channels, and intracellular signal proteins. Facilitative actions of these proteins that regulate efficiency of neurotransmissions in the cerebral cortex circuit may explain the ability of tDCS to induce LTP (Figure 3).

Brain-derived neurotrophic factor (BDNF) may also mediate the development of LTP (Cocco et al., 2018). So far, multi-session anodal simulations on the left dorsolateral prefrontal cortex (DLPFC) has been shown to improve mood symptoms *without* significant change in BDNF concentrations in the blood of patients with major depressive disorder (Brunoni et al., 2015). Further study is warranted to see if tDCS affects BDNF levels in other psychiatric disorders.

Glial cells, including astrocytes, have been reported to be activated by tDCS (Ruohonen and Karhu, 2012). As these cells regulate the concentrations of chemical substances and neurotransmitters in the outer space of neurons, the mechanisms by which tDCS ameliorate psychiatric symptoms may involve some modalities other than direct actions on neuronal cells. For example, findings from animal studies suggest involvement of LTP and glial cells (see Table 5). In rats, anodal tDCS stimulation on hippocampal CA3-CA1 synapses has been reported to induce LTP (Ranieri et al., 2012). Also, tDCS increases cAMP accumulation in the polarized cortex (Hattori et al., 1990), and changes mRNA expressions, leading to the increase in the density of dendritic spines in subjects with strokes (Jiang et al., 2012).

**Table 5:** Animal studies on the mechanism of long-term potentiation (LTP) by anodal tDCS.

**Study**	**Age or weight, species**	**Stimulation intensity, duration**	**Stimulation site**	**Results**
Hattori et al. (1990)	190–240 g Wistar rats	0.3, 3, or 30 μA 30–240 min, 3–5 times per day for several days	Sensory-motor cortex	Increased cAMP accumulation in the polarized cortex in 3 μA. Decreased cAMP accumulation in the polarized cortex in 0.3 μA.
Fritsch et al. (2010)	Male 6–8 weeks old mice	10 μA	Motor cortex (M1) slices	Induced synaptic plasticity *in vitro* by DCS, which was dependent on enhanced BDNF-secretion and TrkB-activation.
Jiang et al. (2012)	Adult, Sprague-Dawley rats, model of middle cerebral artery occlusion	mA, 30 min 3, 7, or 14 sessions	Primary motor cortex	Enhanced density of dendritic spines after stroke. No change in the up-regulated PX1 mRNA expression after stroke. Improved post-stroke motor function on days 7 and 14.
Ranieri et al. (2012)	150–200 g Wistar rats	200–250 μA, 20 min	Hippocampal slices	Modulated LTP at rat hippocampal CA3-CA1 synapses.
Podda et al. (2016)	Male 30–45 days old mice	350 μA, 20 min	Left hippocampus	Exhibited 1-week lasting enhancement in hippocampal LTP, learning, and memory, which were associated with enhanced acetylation of BDNF promoter I, expression of BDNF exons I and IX, and BDNF protein levels. Enhanced CREB phosphorylation, pCREB binding to BDNF promoter I, and recruitment of CREB-binding protein.
Monai et al. (2016)	8–12 weeks old mice	0.1 mA, 10 min	Primary visual cortex	Induced surges of Ca^2+^ influx into astrocytes across the entire cortex by using a transgenic mouse expressing G-CaMP7 in astrocytes. Changed the meta-plasticity of the cortex through astrocytic Ca^2+^/IP3 signaling.
Yu et al. (2019)	8 weeks old male Sprague-Dawley rats	250 μA, 30 min	Hippocampal CA1 slices	Enhanced LTP in hippocampal CA1 slices from rats. Exhibited high levels of BDNF in the hippocampal CA1 region.

Monai et al. reported that tDCS augments noradrenaline levels by increasing intracellular calcium ion concentrations through stimulation of α1 adrenergic receptors on astrocytes in genetically modified mice (Monai et al., 2016). Also, an increase in intracellular concentrations of calcium ions by tDCS has been found in human cells (Dubé et al., 2012), suggesting the involvement of astrocytes in the ability of tDCS to induce LTP.

BDNF binds to TrkB receptors that regulate the growth and synaptic activity of neurons, and are thought to be involved in the formation of LTP (Stephen, 2013). For example, anodal tDCS induces synaptic plasticity *in vitro*, which is dependent on enhanced BDNF-secretion and TrkB-activations (Fritsch et al., 2010). Moreover, Podda et al. (2016) reported that mice subjected to anodal tDCS exhibited hippocampal LTP and improvement of learning and memory. These effects have been reported to be associated with enhancement of acetylation of BDNF promoter I, expression of BDNF exons I and IX, and BDNF protein levels (Podda et al., 2016). The hippocampi of mice receiving tDCS also exhibit enhanced CREB phosphorylation, and phosphorylated CREB at Ser133 (pCREB^133^) binds to BDNF promoter I, and recruits of CREB-binding proteins. These findings suggest that anodal tDCS increases hippocampal LTP and memory via mechanisms related to BDNF genes (Podda et al., 2016; Yu et al., 2019).

## Conclusions

In this review, we discussed the electrophysiological understanding of tDCS on the basis of the stimulation-dependent model. Biochemically, enhancement of excitatory synaptic transmissions through anodal stimulation is likely to facilitate glutamate transmission and suppress gamma-aminobutyric acid transmission in the cortex. Accordingly, tDCS may positively or negatively regulate dopamine, serotonin, and acetylcholine transmissions. These neural events may change the balance between excitatory and inhibitory inputs. In this way, anodal stimulation may modulate activity levels of multiple network systems.

LTP may also provide putative mechanisms underlying the ability of tDCS to treat psychiatric disorders. Future studies should consider other domains of symptoms of psychiatric conditions of schizophrenia and mood disorders, e.g., social cognition and meta-cognition (Nishida et al., 2018; Yamada et al., 2019), as a target of treatment with tDCS. Also, identifications of predictors for its therapeutic benefits in clinical settings deserve further endeavors (Bulubas et al., 2019).

## Limitations

The current review is narrative and the articles were not systematically searched. Moreover, many of the papers presented in this review targeted healthy individuals rather than those with psychiatric disorders. It should be noted that patients with mental illnesses might respond differently to tDCS from healthy people. Furthermore, some articles included in this review were on the effect of tDCS over motor cortex. Further study is warranted to examine evidence for stimulation on prefrontal cortex that has been the main target of psychiatric disorders, such as depression and schizophrenia (Mezger et al., 2020).

## Author Contributions

YY and TS planned and designed the review, made substantial contributions, and approved the final manuscript. YY collected the data and drafted the first manuscript. TS critically reviewed the draft and revised it. All authors contributed to the article and approved the submitted version.

## Conflict of Interest

The authors declare that the research was conducted in the absence of any commercial or financial relationships that could be construed as a potential conflict of interest.
